# Synthesis and characterization of a new copper-based polyoxomolybdate and its catalytic activity for azide-alkyne cycloaddition reaction under UV light irradiation

**DOI:** 10.1038/s41598-023-50624-0

**Published:** 2024-01-05

**Authors:** Mojtaba Amini, Asmaa Yousofvand, Mojtaba Hosseinifard, Arshad Bayrami, Jan Janczak

**Affiliations:** 1 Department of Inorganic Chemistry, Faculty of Chemistry, University of Tabriz, Tabriz, Iran; 2 Department of Physical Chemistry, Faculty of Chemistry, University of Tabriz, Tabriz, Iran; 3 Department of Energy, Materials and Energy Research Center, Karaj, Iran; 4 Department of Chemistry, Research Center for Development of Advanced Technologies, Tehran, Iran; 5 Institute of Low Temperature and Structure Research, Polish Academy of Sciences, Okólna 2 Str., 50-422 Wrocław, Poland

**Keywords:** Catalysis, Materials science

## Abstract

A new organic-functionalized Cu-based Anderson-type polyoxomolybdate, namely (C_7_H_15_N_4_)_2_[Na(H_2_O)_4_]_2_[C_6_H_12_CuMo_6_N_2_O_24_]·2(H_2_O) (Cu^II^-POM), was synthesized via a simple one-pot reaction and subsequently characterized using a range of analytical and spectral techniques. Structural investigation by single crystal X-ray diffraction analysis revealed that the polyanion component of the synthesized compound (i.e. [C_6_H_12_CuMo_6_N_2_O_24_]^4−^) possesses a δ-isomer Anderson-type structure, which is surrounded by four lattice water molecules and four [C_7_H_15_N_4_–NaH_15_(H_2_O)_8_]^4+^ cations in the crystal packing arrangement. The resulting double-sided tris-functionalized Anderson-type compound can function as highly effective heterogeneous photocatalysts for the copper(I)-catalyzed Huisgen azide-alkyne cycloaddition (Cu-AAC) reaction of terminal alkyne, benzyl halides, and sodium azide (acts as the azidonation and reducing agent) in aqueous media. Ultraviolet light irradiation enhances the catalytic activity of Cu^II^-POM ~ 4.4 times of the “off” situation under reaction conditions of 0.00239 mmol cat., 80 °C, 8 h, 2 mL H_2_O, So that the isolated yields for the AAC reaction involving a variety of terminal alkynes and benzyl halides using the Cu^II^-POM catalyst ranged between 19–97%. The current study is the first report about using an efficient and economical Cu(II)-POM/UV/NaN_3_ catalytic system in the Cu-AAC reaction and reveals its significant potential for applying to other Cu(I)-catalyzed reactions.

## Introduction

Today, research in the field of utilizing cost-effective and eco-friendly catalysts has expanded. In the meantime, the diverse uses of polyoxometalates (POMs) have gained significant attention due to their exceptional stability, facile synthesis, and nanostructural properties^1,2^. POMs consist of metal ions and oxides, where the oxides act as ligands by sharing an additional pair of electrons via metal bridging and polyhedral bonding, forming closed and extensive three-dimensional (3D) structures. The employed primary metal ions in POM clusters production included group VI and group V elements in higher oxidation states with d0 or d1 electron structures, which render polyoxometalates versatile for diverse applications^3–12^. Some of these applications include the synthesis and stabilization of nanoparticles as catalysts for the design and synthesis of some materials^8^, energy storage^10^, electrochemical sensors^13–15^, catalysts and photo/electrocatalysts^16–24^ etc.

Photocatalytic degradation of toxic organic pollutants and unpleasant and non-biodegradability coloring compounds from water, as well as photo-reduction application and recovery of valuable metal ions and heavy metal ions removal, are the recently attended POMs^25–28^. Although efforts have been made to shift the absorption band of POM to the visible light region, most of these compounds become active under ultraviolet (UV) light irradiation^29–32^. As extensively detailed in former literature, UV light irradiation on POMs causes an oxygen-to-metal charge transfer (O → M CT) as a result of promoting an electron from the highest occupied molecular orbital (HOMO) to the lowest unoccupied molecular orbital (LUMO). The resulting photo-excited POMs, known as POMs*, are potent oxidizing compounds and are able to form POM^−^ through the abduction of an electron from proper reducing compounds^27,33^. The as-prepared POM^−^ has the ability to deliver its electrons to various chemical species, for instance, metal ions.

Functionalization of POM with organic ligands is an appropriate method to extend the structure of this group of compounds and control their electronic properties, stability, and compatibility with organic media^34,35^. Among the various synthetic strategies carried out in this field (organoalkoxylation, organoimidization, organoarsonylation, organosilylation, organophosphonylation and organotin), unique interest has been paid to the covalent attachment of organic moieties to the POM surface in direct mode (organoimidization and organoalkylation)^34,36^. Apart from other advantages of the organoalkylation strategy (e.g. more effective synthesis), the greater stability of organoalkylated POMs in aqueous media compared to organoimidizated counterparts can make them susceptible to use in water-active catalytic systems^36,37^. Anderson-type POMs are one of the well-studied compounds for grafting strategies with a wide variety of multidentate alkoxy ligands (such as tris-ligands; RC(CH_2_OH)_3_, R = –NH_2_, –OH, –CH_3_ etc.). To date, numerous isomers of single-side and double-side tris-functionalized structures have been prepared and reported from them^38–42^. The δ-isomer of Anderson-type POM is one of the tris-functionalized Anderson derivatives, in which triol ligands replaced all three μ^3^-OH oxygen atoms at one sides of the flat POM cluster. These compounds are typically produced in several complex steps, and alternative and attractive one-step production methods require further research.

The Cu(I)-catalyzed Huisgen cycloaddition of azides and alkynes, also known as Cu-AAC, is the most well-known click reaction that produces 1,2,3-triazoles gently and selectively. Triazoles are relatively stable compounds with numerous applications in medicine, organic chemistry, and bioregional synthesis^43–46^. Furthermore, triazole derivatives such as fluconazole have been synthesized and designed as antifungal agents^47^. Thus far, various catalytic systems containing a wide range of copper species, such as Cu(II) compounds with a reducing agent (typically sodium ascorbate) or Cu(I) compounds, have been developed for catalyzing AAC reactions^48,49^. Lately, the photo-chemically conducted Cu-AAC reactions under UV light irradiation (the strategy of in situ generation of Cu^I^ from a Cu^II^ using light) have demonstrated their potential as a dependable approach for diverse triazoles synthesis applications^50–52^. Cu(I) generation during the light-induced Cu-catalyzed reactions can be accomplished by either directly exposing Cu(II) compounds to UV and vis irradiation or indirectly reducing Cu(II) using a photosensitizer^51^. In the direct photoreduction of Cu(II) centers, promoting an intramolecular electron transfer from the ligand’s π-system to the Cu(II) center and forming Cu(I) species occurs through UV light absorption. In the commonly used indirect strategy, the photoinitiator first absorbs ultraviolet light and forms a reaction intermediate, which can then promote the photoreduction of Cu(II) to Cu(I) species^53,54^. Nevertheless, to our knowledge, there is no report on utilizing UV light-assisted photocatalytic Cu-AAC reaction based on Anderson-type POMs, where no supplementary reducing agent or photoinitiator is needed.

Here, a new Cu-based δ-isomer Anderson-type polyoxometalate (Cu^II^-POM) was synthesized, and after fully structural characterization, it was used as an efficient catalyst in the Cu-AAC reaction to prepare target 1,2,3-triazoles under UV light irradiation without the need for an additional reducing agent. The stability and true heterogeneity of the as-prepared catalyst were also assessed under previously established optimal reaction conditions.

## Experimental

### One-pot synthesis of Cu-based δ-isomer Anderson-type polyoxomolybdate (C_7_H_15_N_4_)_2_[Na(H_2_O)_4_]_2_[C_6_H_12_CuMo_6_N_2_O_24_]·2(H_2_O) (Cu^II^-POM)

5.85 mmol of sodium molybdate dihydrate was added to a mixed solution of 15 mL acetic acid, 2 mL methanol, and 25 mL deionized (DI) water and stirred for 5 min. To this solution, copper nitrate trihydrate (2.06 mmol) and ammonium acetate (12.97 mmol) were added, and the resulting mixture was agitated at 70 °C for a duration of 4 h. Finally, the solution (in an open vial) was positioned in a fixed place for crystal formation. The combined yield of both crystals and bulk powder is 65%.

### General procedure for the Cu-AAC under UV light irradiation

Terminal alkyne (0.5 mmol), sodium azide (0.55 mmol), and the organic halide (0.55 mmol) were added to 2 mL H_2_O containing 0.00239 mmol Cu^II^-POM as a catalyst. Then, the reaction mixture was warmed to 80 °C in an oil bath and stirred under UV light irradiation. Following a specific duration, the resulting mixture was extracted with ethyl acetate (EtOAc; 15 mL), and the gathered organic phase was dried over anhydrous CaCl_2_. After removing the solvent under reduced pressure, it was possible to acquire the target 1,2,3-triazoles without any requirement for a purification step. Ultimately, the obtained product was weighed and the reaction efficiency was calculated. The ^1^HNMR technique was used for product validation.

## Results and discussion

### Characterization of Cu^II^-POM

**Figure 1:**
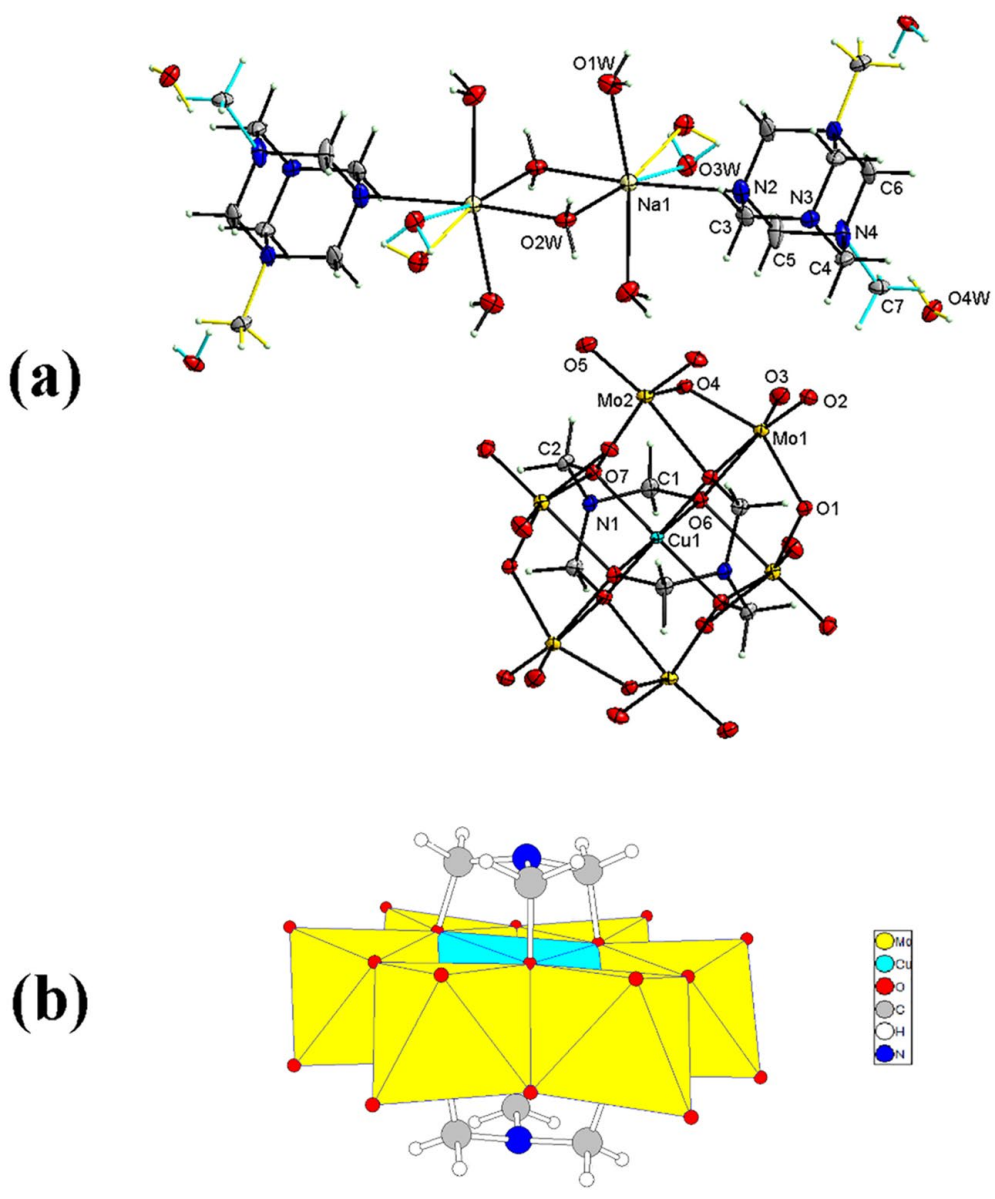
(**a**) View of the Cu^II^-POM, with the labelling of the atoms. Thermal ellipsoids are depicted at the 50% probability level, H atoms with arbitrary radii. The site occupancy factors of the two disordered parts were refined to 0.5 (turquoise and yellow). (**b**) Polyhedral representation of the [C_6_H_12_CuMo_6_N_2_O_24_]^4−^ in the crystal structure.

The double-sided symmetric tris-functionalized Anderson-type POM with a copper(II) heteroatom and the Mo(VI) addenda atoms (Cu^II^-POM) was prepared via a one-pot reaction protocol. In this facial approach, the addenda atom, the heteroatom, and ammonium acetate salts were dissolved in acetic acid/methanol/DI water mixed solution and heated at 70 °C for 4 h to produce the desired tris-functionalized δ-isomer Anderson cluster. The resulting compound is sufficiently stable to be preserved in air for several months. Also, the solubility test revealed that the Cu^II^-POM is insoluble in acetone, ethyl acetate and water but it is barely soluble in ethanol, methanol, and acetonitrile. Single-crystal X-ray analysis uncovered that (C_7_H_15_N_4_)_2_[Na(H_2_O)_4_]_2_[C_6_H_12_CuMo_6_N_2_O_24_]·2(H_2_O) crystallizes in a monoclinic space group *I2/m.* The detailed crystal structure of Cu^II^-POM is depicted in Fig. 1. The anion of [C_6_H_12_CuMo_6_O_24_]^4−^ possesses the conventional flat Anderson-type polyoxomolybdate structure, consisting of an edge-sharing central heteroatom octahedra unit (CuO_6_) surrounded by six edge-sharing MoO_6_ octahedra units. Two trimethanolamine (tris) ligands, which are produced during the reaction, were attached to the cluster by supplanting six μ_3_-oxygen atoms on either side of the plane to form a δ-isomer of the tris-functionalized Cu-based Anderson-type POM. Whereas the copper atom is connected to six deprotonated μ_3_-oxygens from the tris ligand, each molybdenum atom is encompassed by two terminal oxygens, two μ_2_-bridged oxygens, and two μ_3_-oxygens of the tris ligand. Within the cation of [C_14_H_46_N_8_Na_2_O_8_]^4+^, each sodium atom is surrounded by one N-methyluroptropine (C_7_H_15_N_4_)^+^ molecule and five water molecules (two bridged water and three terminal water). The methyl group on the N-methyluroptropine (C7, H7A, H7B, and H7C), a lattice water molecule (O4W, H4WA, and H4WB), the oxygen atom of one terminal water molecule (O3W) and the hydrogen atoms of the C1 and C3 (H1A, H1B, H3A, and H3B) each were found to be disordered over two positions and the site occupancy factors were refined to 0.5 in all cases (turquoise and yellow). The particulars regarding the data collection parameters, crystallographic data, and final agreement parameters have been compiled in Table 1.

**Table 1:** Crystal data, data collection, and structure refinement details for Cu^II^-POM.

Parameters	Cu^II^-POM
Empirical formula	(C_7_H_15_N_4_)_2_[Na(H_2_O)_4_]_2_[C_6_H_12_CuMo_6_N_2_O_24_]·2(H_2_O)
Formula weight (g mol^−1^)	1671.95
Crystal system, space group	Monoclinic, I2/m
α = γ, β (°)	90, 98.149(1)
a, b, c (Å)	8.4704 (3), 13.2997 (5), 21.9579 (8)
V (Å^3^)	2448.29 (16) Å^3^
Z	2
D_calc_ (g·cm^−3^)(D_x_)	2.234
µ (mm^−1^)	2.04
F(000)	1654
Crystal size (mm)	0.28 × 0.26 × 0.24 mm
Radiation type, wavelength, λ (Å)	Mo *Kα* radiation, 0.71073 Å
Temperature (K)	100
θ range (°)	3.1–29.0°
Absorption correction	Multi-scan
T_min_/T_max_	0.893/1.000
Reflections collected / unique / observed	25,906/3288/2946
Reflections/ parameters/ restraint	3288/208/5
R_int_	0.029
L.S. parameters	208
Refinement on	F^2^
R[F^2^ > 2σ(F^2^)]	0.022
wR(F^2^ all reflections)^a^	0.051
Goodness-of-fit, S	1.00
∆ρ_max_, ∆ρ_min_ (e Å^−3^)	1.76 e Å^−3^, − 0.82 e Å^−3^
(Δ/*σ*)_max_	0.002

^a^*w*^−1^ = [σ^2^(*F*_o_^2^) + (*aP*)^2^ + (*bP*)] where *P* = (*F*_o_^2^ + 2*F*_c_^2^)/3. The *a* and *b* parameters are 0.021 and 8.3078.

In the polyanion [C_6_H_12_CuMo_6_O_24_]^4−^ the distances from the copper to alkoxo oxygen atoms (Cu–O bonds) are from 1.9705 (14) to 2.224 (2) Å. On the other hand, the average bond lengths of the bridged, terminal, and alkoxo oxygens with molybdenum are 1.9347, 1.7133, and 2.2690 Å (range from 1.9264 (10) to 1.9417 (14), 1.7074 (15) to 1.7180 (15), and 2.2207 (13) to 2.3348 (14) Å. The O–Cu–O bond angles around the Cu center, vary from 84.45° (8) to 180.0° (7), and the hexakis-alkoxo coordinated Cu(II) ion (CuO_6_) exhibits a distorted octahedral coordination sphere. Selected bond lengths [Å] and angles [°] for the polyanion part of Cu^II^-POM are provided in Supplementary Table S1 online.

A view of the crystal packing of the Cu^II^-POM in the direction of the a-axis is depicted in Supplementary Fig. S1 online. As it evident, each [C_6_H_12_CuMo_6_O_24_]^4−^ polyanion is surrounded by four lattice water molecules and four [C_7_H_15_N_4_–NaH_15_(H_2_O)_8_]^4+^ cations to form columns along the a-axis direction. Supplementary Table S2 online lists efficient interactions (hydrogen bonding) for building such a framework structure.

The composition of the prepared POM was also further characterized by the energy-dispersive X-ray spectroscopy (EDX) elemental analyses, inductively coupled plasma optical emission spectrometry (ICP-OES), carbon, hydrogen and nitrogen (CHN) elemental analysis as well as FT-IR spectroscopy. The EDX analysis results confirmed the presence of N, C, O, Cu, and Mo elements in the structure of the prepared Cu^II^-POM, in accordance with the results of single-crystal X-ray analysis (see Supplementary Fig. S2 online). The ICP and CHN results closely match the theoretically calculated values for CuII-POM, with the following percentages: calcd (exptl); C 14.37 (13.77), H 3.74 (4.05), Cu 3.80 (3.64), Mo 34.43 (33.01), N 8.38 (8.03).

Figure S3 shows the TGA of the Cu^II^-POM from 25 to 1000 °C. The Cu^II^-POM showed the initial low-temperature weight loss of 3.8 wt% due to water molecules. Then, the weight loss (21%) beginning at 210 °C is primarily due to the decomposition of N-methyluroptropine (C_7_H_15_N_4_)^+^ cations, indicating that the Cu^II^-POM is stable below 210 °C. The weight loss of Cu^II^-POM with a mild slope occurred between 210 and 650 °C corresponds to the proportion of trimethanolamine (tris) ligands and oxygen molecules contained in the Cu^II^-POM (19 wt%).

**Figure 2:**
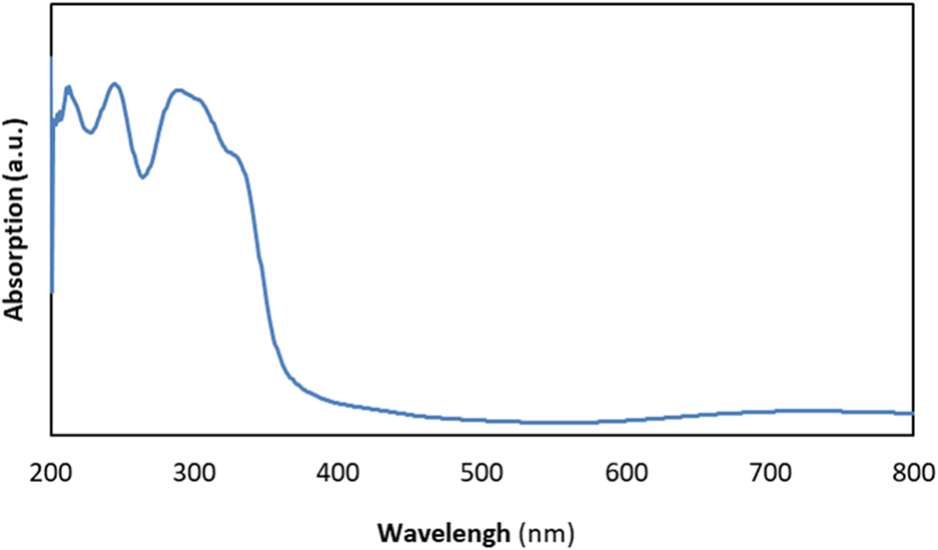
Diffuse reflectance spectrum of the as-synthesized Cu^II^-POM.

In the FT-IR spectrum of Cu^II^-POM, the characteristic bands of water, N-methyluroptropine, and tris are observed at 3100–3700 (the broad band with the center at 3500 cm^−1^), 3004, 2924, 2860, 1466, 1255, and 1020 cm^−1^ resulting from *ν* O–H of water molecules, *ν* C–H of CH_3_, *ν*_assym_ C–H, *ν*_sym_ C–H, *δ* C–H of CH_2_, and *ν* C–N (on tris and N-methyluroptropine), respectively (see Supplementary Fig. S4 online)^55^. Moreover, the peaks located at 901. 828, and 645 cm^−1^ are related to Mo=O_t_, Mo–O_b_–Mo, and Cu–O vibration modes, confirm the successful synthesis of desired POM. Additionally, the powder X-ray diffraction (PXRD) pattern (see Supplementary Fig. S5 online) of the as-synthesized Cu^II^-POM is nearly similar to the simulation result of single crystal (C_7_H_15_N_4_)_2_[Na(H_2_O)_4_]_2_[C_6_H_12_CuMo_6_N_2_O_24_]·2(H_2_O) (especially in the 2θ below 20), indicating the same chemical structure with lower crystallinity of the bulk powder. The ultraviolet–visible (UV–Vis) diffuse reflection spectroscopy of the yellowish-green solid sample of Cu^II^-POM exhibited three strong absorptions in the ultraviolet region (up to approximately 385 nm), indicating its potential application in UV-activated photocatalysis (Fig. 2).

### Catalytic effects

Catalytic activity assessment began with the cycloaddition of phenylacetylene (0.5 mmol) to in situ-generated benzyl azide [from sodium azide (0.55 mmol) and benzyl chloride (0.55 mmol)] in the presence of Cu^II^-POM catalyst. All the catalytic reactions were conducted under atmospheric and aqueous reaction conditions and direct exposure to UV light irradiation (on/off situation). The optimal conditions were determined by varying the parameters affecting the reaction rate, like solvents, temperature, reaction duration time, and catalyst nature (Table 2). The results showed that without utilizing a catalyst, a control experiment, the intended triazole product cannot be produced even under UV light irradiation (entry 1). Whereas, by loading a small amount of Cu^II^-POM catalyst (0.00239 mmol) at 25 °C, the yield of the isolated product under UV light enhanced to 63% after 8 h (entry 6). Further enhancement in catalytic efficiency was achieved by raising the reaction temperature, as the molecules’ higher velocity increases the frequency of their possible collisions. The maximum product yield was obtained in 8 h at a temperature of 80 °C (entries 6, 9–14) and raising the temperature even more has no impact on the reaction's progress (entry 13). Altering the reaction duration time also had a remarkable influence on the reaction rate (entries 2–6). As shown in Table 2, increasing reaction time from 2 to 8 h raises the yield from 75 to 97% under UV light in “on” situation. Increased reaction duration to 10 h does not enhance the reaction efficiency in either the “on” or “off” states (entry 14). In addition, it was found the efficiency of the reaction was strongly affected by the solvent type (entries 6, 15–18). Several different solvents, including acetonitrile, water, acetylacetone, ethanol, and methanol were evaluated in order to determine the best solvent for the model reaction. No significant product was observed when CH_3_OH, C_2_H_5_OH, and C_3_H_6_OH were used as solvents, while the addition of CH_3_CN and H_2_O enhanced the product yield to 78% and 97%, respectively. The convenient solubility of sodium azide in water is the main reason for this observation. Comparison of the catalytic potential of the as-fabricated POM with its parent salts revealed the superior performance of Cu^II^-POM catalyst at the same reaction conditions, where Cu(NO_3_)_2_·3H_2_O and Na_2_MoO_4_·2H_2_O produce 63% and 87% of desired triazole product under UV light irradiation. Additionally no synergistic catalytic effect between the raw materials Cu(NO_3_)_2_·3H_2_O and Na_2_MoO_4_·2H_2_O on reaction efficiency was observed (entry 9).

**Table 2:** The outcomes for various circumstances on the azide-alkyne click reaction.

Entry	Catalyst	Solvent	Temperature (°C)	Time (min)	Yield (%)^a^ UV light	
					On	Off
1	–	H_2_O	80	480	0	0
2	Cu^II^-POM	H_2_O	80	120	75	22
3	Cu^II^-POM	H_2_O	80	180	82	22
4	Cu^II^-POM	H_2_O	80	240	90	17
5	Cu^II^-POM	H_2_O	80	360	94	16
6	Cu^II^-POM	H_2_O	80	480	97	22
7	Cu(NO_3_)_2_·3H_2_O	H_2_O	80	480	63	71
8	Na_2_MoO_4_·2H_2_O	H_2_O	80	480	87	68
9	Cu(NO_3_)_2_·3H_2_O and Na_2_MoO_4_·2H_2_O	H_2_O	80	480	66	59
10	Cu^II^-POM	H_2_O	25	480	63	15
11	Cu^II^-POM	H_2_O	40	480	82	31
12	Cu^II^-POM	H_2_O	60	480	88	36
13	Cu^II^-POM	H_2_O	100	480	89	24
14	Cu^II^-POM	H_2_O	100	600	91	14
15	Cu^II^-POM	C_2_H_5_OH	80	480	24	–
16	Cu^II^-POM	CH_3_OH	80	480	30	–
17	Cu^II^-POM	C_3_H_6_OH	80	480	41	–
18	Cu^II^-POM	C_2_H_3_N	80	480	78	–

Reaction conditions: catalyst (0.00239 mmol), benzyl chloride (0.55 mmol), phenylacetylene (0.5 mmol), sodium azide (0.55 mmol), and solvent (2 mL). ^a^Isolated yields.

**Figure 3:**
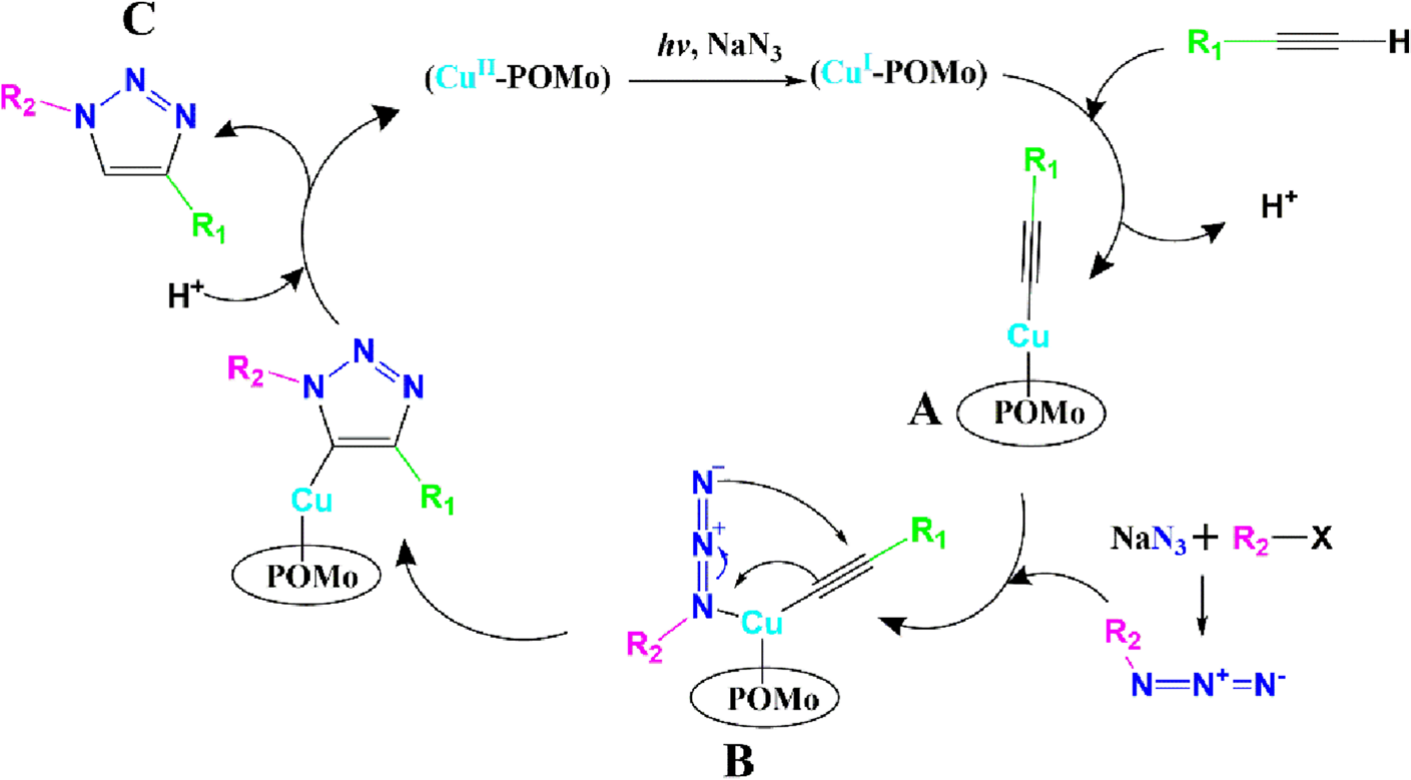
Proposed mechanism for the AAC reaction in the presence of Cu^II^-POM under exposure to UV light irradiation.

Based on our experimental results and the related literature studies, a possible reaction mechanism is proposed, as depicted in Fig. 3. As mentioned early (see the introduction section), POM compounds have the ability to form an excited state POM* (Cu^II^-POM* here) with robust oxidizing power as a consequence of charge transfer (CT) from O^2−^ to Mo^6+^ (the O → Mo CT) under exposure to UV light. The photoexcited POM* can abstract electrons from a wide range of compounds, for instance, propan-2-ol, and form a photochemically reduced POM^−^ (Cu^II^-POM^−^ here), which then reduces its Cu(II) centers through a metal-to-metal CT (Mo^V^ → Cu^II^) to create a favorable Cu(I) site for catalyzing the AAC reaction (Cu^I^-POM formation)^56^. Whereas alcoholic solvents are known as sacrificial reducing reagents in metals photocatalytic reduction and recovery studies^27,28^ they are not favorable for our catalytic system, as can be seen from Table 2 (entries 12–14). On the other hand, by conducting a reaction between phenylacetylene benzyl azide and in the absence of NaN_3_, only 15% of the target 1,2,3-triazole was achieved. These results are consistent with former documents about the in situ generation of Cu(I) species in the presence of NaN_3_ and disclose both the reducing and azidonation property of the sodium azide in our developed catalytic system^57^. In the continuation of the catalytic cycle, the newly generated Cu(I) sites attack alkyne to produce the Cu(I)-acetylide intermediate, **A**. Subsequently, the in situ formed azide molecules (from NaN_3_ and benzyl chloride reactants) attach to this complex to generate an intermediate complex of **B**. The nucleophilic attack of nitrogen of the azide to alkyne forms copper(I) metallacycle, which can create the target product (**C**) after the protonation and elimination of the catalyst. Performing the model reaction in a dark condition (with a yield of 35%) indicated that the reaction could not proceed entirely without UV light irradiation exposure, supporting the proposed mechanism here.

Table 3 illustrates the scope of terminal alkynes and benzyl halides in the AAC reaction under UV light irradiation using the Cu^II^-POM catalyst. Under optimized reaction conditions (0.00239 mmol cat., 80 °C, 8 h, 2 mL H_2_O, and UV light irradiation), aromatic alkyne of phenylacetylene demonstrated higher reactivity than aliphatic alkynes, and the lowest product yield (19%) belongs to the 2-methyl-3-butyn-2-ol substrate with the highest number of methyl groups (steric effect). The nature of the leaving group on benzyl halide (Cl and Br) does not significantly affect the reactivity. In contrast, the substitution of benzyl chloride with a nitro-withdrawing group at the ortho-position decreased the yield of desired 1,2,3-triazoles.

**Table 3:** Cu^II^-POM-catalyzed cycloaddition reactions of the substituted benzyl halides and phenylacetylenes with NaN_3_ under UV light irradiation.


Entry	Alkyl halide	Terminal alkyne	Product	Yield (%)^a^
1				97
2				48
3				94
4				71
5				80
6				19
7				41

Reaction conditions: alkyl halide (0.55 mmol), terminal alkyne (0.5 mmol), sodium azide (0.55 mmol), catalyst (0.00239 mmol), and H_2_O (2 mL). ^a^Isolated yields.

To reveal the competency of the Cu^II^-POM catalyst, the AAC reaction results under the optimal reaction conditions in the current study were compared to former studies and summarized in Supplementary Table S3 online. Although our results are comparable to other data presented in this table, using an easy and fast synthesis method (facial one-pot synthesis protocol) without any need for substrate material for providing heterogeneous nature (compared to entries 4 and 5), performing the catalytic reaction in green media (in contrast to entries 2 and 4), low catalyst loading, fewer reaction time (in comparison to 1–2, 5), and no need for additives (like the base and ligand in entries 2–3) are the major advantages of our described catalytic system—indicating the high potential of our designed Cu^II^-POM catalyst for 1,2,3-triazoles production. Additionally, the photocatalytic approach employed in this study is the novelty of the work, which shows promise for advancing research in this field and generating further interest.

**Figure 4:**
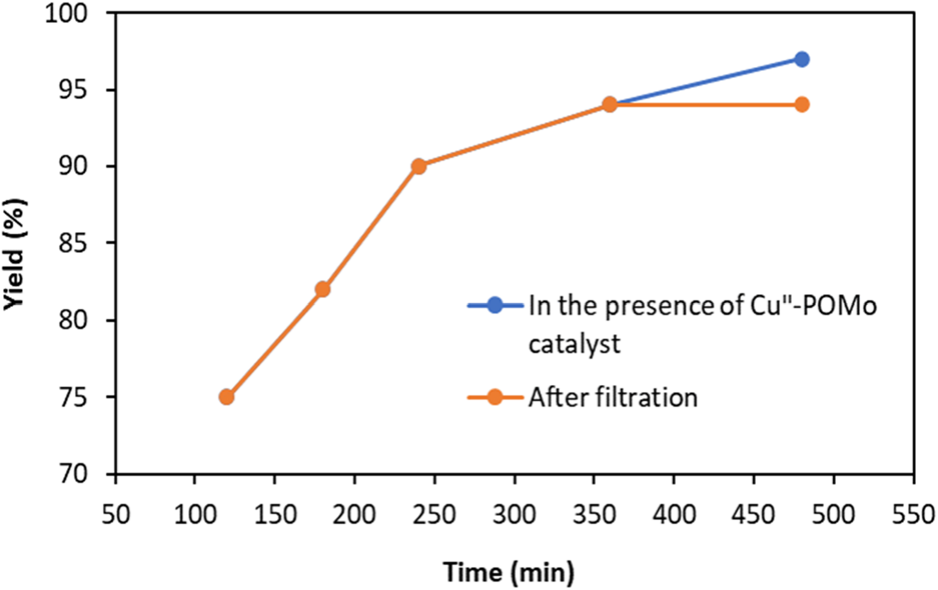
Hot filtration test for the CuII-POM-catalyzed AAC reaction.

The hot filtration experiment of Cu^II^-POM has been carried out to certify its actual heterogeneous catalytic activity (Fig. 4). In this regard, the Cu^II^-POM catalyst was recovered by hot filtration after 6 h, and the catalytic reaction was further performed in the catalyst-free aquatic phase (the filtrate). The results showed that no appreciable enhancement in the cycloaddition efficiency is achieved after 2 h, suggesting no metal active sites leaching and the factual heterogeneity of the as-synthesized catalyst.

**Figure 5:**
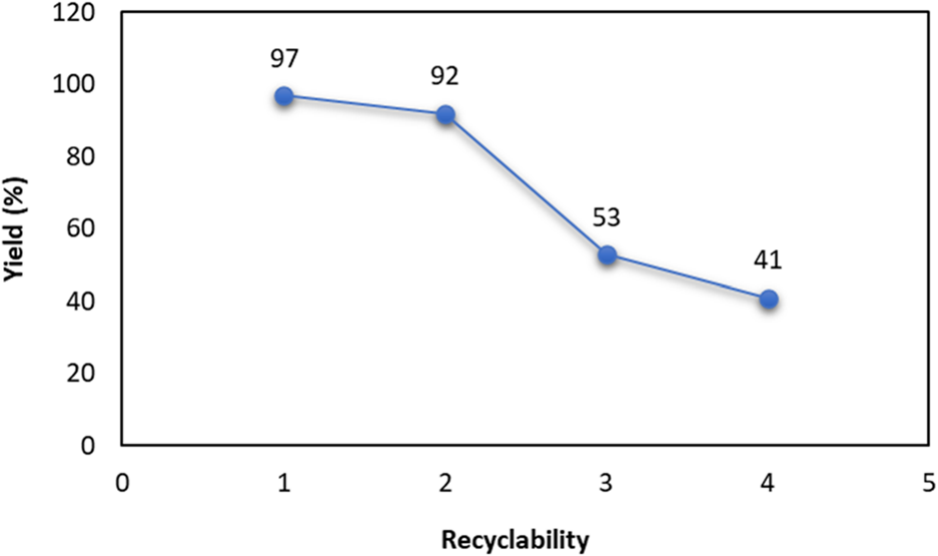
Recycling studies of the Cu^II^-POM catalyst in the AAC reaction.

Furthermore, the recyclability of the as-fabricated Cu^II^-POM has been checked under the optimized reaction conditions (Fig. 5). For this purpose, after extraction of the desired triazole product (using 2 × 5 mL of EtOAc), the catalyst was isolated from the aqueous phase by centrifugation, washed by water and ethanol, dried at 60 °C, and then used in the subsequent batch of reactions by adding fresh substrates. It was found that the activity of the Cu^II^-POM catalyst is maintained for two initial cycles, after which a significant drop in activity was observed for two further successive cycles (product yield decreased to 41%). Based on the PXRD and FT-IR findings of the fresh and used photocatalyst (analyzed after three runs), it is evident that a structural transformation to a new inactive crystalline form is the primary cause for this observed phenomenon (see Supplementary Figs. S4 and S5 online).

## Conclusion

In conclusion, a facial and economical one-pot protocol was developed for the synthesis of a double-sided tris-functionalized Anderson-type Cu^II^-POM catalyst from its parent salts. The as-synthesized catalyst was thoroughly characterized using single crystal X-ray diffraction, PXRD, ICP, CHN, UV–Vis, TGA, EDX and FT-IR analysis, utilized as an efficient catalyst in 1,2,3-triazoles synthesis. The catalytic reaction results demonstrated that our fabricated catalyst is only active when exposed to UV light and performs in water without any requirement for additional reducing agents. Additionally, a hot filtration and recyclability test verified its excellent stability and moderate reusability under applied media. The outcome of this research demonstrates in situ generation of Cu(I) from UV-activated Cu(II)-based POMs is a proper method for one-pot catalytic production of 1,2,3-triazoles.

### Supplementary Information

Supplementary Information.

## Data Availability

Full details of the X-ray data collection and final refinement parameters, including anisotropic thermal parameters and a full list of the bond lengths and angles, have been deposited with the Cambridge Crystallographic Data Center in the CIF format as supplementary publications no. CCDC 2284167. Copies of the data can be obtained free of charge on the application to CCDC, 12 Union Road, Cambridge, CB21EZ, UK, (fax: (+44) 1223-336-033; email: deposit@ccdc.cam.ac.uk). The PXRD patterns, TGA plot, SEM–EDX and FT-IR spectra of the Cu^II^-POM along with the table for the comparison of the results with the literature are available in the Supplementary Information file.
