# Supplementary material for: Synthesis and characterization of a new copper-based polyoxomolybdate and its catalytic activity for azide-alkyne cycloaddition reaction under UV light irradiation
Source: Sci Rep. 2024 Jan 5;14:653. doi: 10.1038/s41598-023-50624-0 (PMC10770156; doi:10.1038/s41598-023-50624-0)
Supplement: Supplementary file 1 — Supplementary Information. [file 41598_2023_50624_MOESM1_ESM.docx]

**Supporting Information**

**Synthesis and characterization of a new copper-based polyoxomolybdate and its catalytic activity for azide-alkyne cycloaddition reaction under UV light irradiation**

Mojtaba Amini^a,*^, Asmaa Yousofvand^b^, Mojtaba Hosseinifard^c^, Arshad Bayrami^d^, Jan Janczak^e^

^a^ *Department of Inorganic Chemistry, Faculty of Chemistry, University of Tabriz, Tabriz, Iran*

*^b^ Department of Physical Chemistry, Faculty of Chemistry, University of Tabriz, Tabriz, Iran*

*^c^* *Department of Energy, Materials and Energy Research Center, Karaj, Iran*

*^d^Department of Chemistry, Research Center for Development of Advanced Technologies, Tehran, Iran*

*^e^Institute of Low Temperature and Structure Research, Polish Academy of Sciences, Okólna 2 str., 50-422 Wrocław, Poland*

* Corresponding authors; Tel.: +98 041 33393130; Fax: +98 41 33390191. Email address: [mojtaba_amini@tabrizu.ac.ir](mailto:mojtaba_amini@tabrizu.ac.ir)

## **Materials and Methods**

Ammonium acetate (NH_4_CH_3_CO_2_), copper nitrate trihydrate (Cu(NO_3_)_2_·3H_2_O), and sodium molybdate dihydrate (Na_2_MoO_4_·2H_2_O) were purchased from Merck. Anhydrous acetic acid (CH_3_CO_2_H) and methanol (CH_3_OH) were prepared by the Iranian Vision Pars Delta Company. Energy dispersive spectrometry (EDX) spectrum was acquired using an EDAX Pegasus XM4 spectrometer with an SDD Apollo 4D detector mounted on an FEI Nova NanoSEM 230 microscope. FT-IR spectrum was obtained as KBr pellets on a Perkin-Elmer 100 FTIR spectrometer over 450–4000 cm^-1^. Elemental analysis was carried out using an ICP-OES Varian 730-ES and Carlo ERBA Model EA 1108 elemental analyzer. Powder X-ray diffraction (PXRD) patterns were characterized with a PANalytical XPert Pro MPD and diffractometer with Cu Kα radiation (λ = 0.154 nm). The UV−vis diffuse reflection data were recorded at room temperature using a powder sample with BaSO_4_ as a standard (100% reflectance) on a Avantes AvaSpec-2048 spectrophotometer and scanned from 200 to 1100 nm. Thermal stabilities were investigated by a

Linseis STA PT1600 thermal analyzer in N_2_ atmosphere with a heating rate of 10 °C/min under N_2_ atmosphere. The employed UV light source was a Philips TUV 15W G15T8 with spectral peak at 254 nm, which was made in Holland.

**Single crystal X-ray data collection**

Synthesis and characterization study by X-ray single crystal [C_6_H_12_CuMo_6_N_2_O_24_] (C_14_H_46_N_8_Na_2_O_8_)·2(H_2_O) (**1**) at RT (295 K) and LT (100 K) temperatures using graphite monochromatic MoKα radiation on a diffractometer Four circular KUMA KM-4 with two-dimensional CCD area. There are no structural phase transitions between RT and LT. Only the temperature factors of the atoms are much smaller in LT. Structural analysis data were measured for these crystals at 100 K. Data collections were made using the CrysAlis CCD program [1]. Integration, scaling of the reflections, correction for Lorenz and polarisation effects, and absorption corrections were performed using the CrysAlis Red program [1]. The structure was solved by the direct methods using SHELXT-2014/7 [2]. and refined using SHELXL-2018/3 program [3]. The positions of hydrogen atoms were introduced in their geometrical positions and treated as rigid. The final difference Fourier maps showed no peaks of chemical significance. Details of the data collection parameters, crystallographic data and final agreement parameters are collected in Table 1. Visualizations of the structures were made with the Diamond 3.0 program [4].

Table S1. Selected bond lengths [Å] and angles [°] for Cu^II^-POM.

| Bond lengths (Å) | | Bond angles (°) | | | |
| --- | --- | --- | --- | --- | --- |
| Mo1-O1 | 1.9264 (10) | O1—Mo1—O6 | 72.02 (6) | O4—Mo1—O6 | 84.55 (6) |
| Mo1-O2 | 1.7180 (15) | O1—Mo1—O4 | 148.34 (7) | Mo2—O4—Mo1 | 118.44 (7) |
| Mo1-O3 | 1.7074 (15) | O2—Mo1—O1 | 95.34 (7) | Mo1—O6—Cu1 | 97.15 (6) |
| Mo1-O4 | 1.9417 (14) | O2—Mo1—O6 | 160.61 (7) | Cu1—O7—Mo2 | 101.91 (6) |
| Mo1-O6 | 2.2207 (13) | O3—Mo1—O2 | 104.87 (8) |  |  |
| Cu1-O6 | 2.224 (2) | O3—Mo1—O1 | 104.13 (8) |  |  |
| Cu1-O7 | 1.9705 (14) | O3—Mo1—O6 | 92.68 (8) |  |  |

Table S2. Hydrogen-bond geometry (Å, º)

| D-H…A | D-H | H….A | D…A | D-H…A |
| --- | --- | --- | --- | --- |
| O_1_W-H_2_W_2_…O_4_^i^ | 0.89 | 1.99 | 2.858(2) | 162 |
| O_2_W-H_2_W…O_5_^i^ | 0.89(1) | 1.98(1) | 2.8109(19) | 171(3) |
| O_3_W-H1W_3_…N_3_^ii^ | 0.85(1) | 2.22(1) | 2.960(4) | 145(1) |

Symmetry cods: (i) x, -y+1, z; (ii) x-1, y, z.


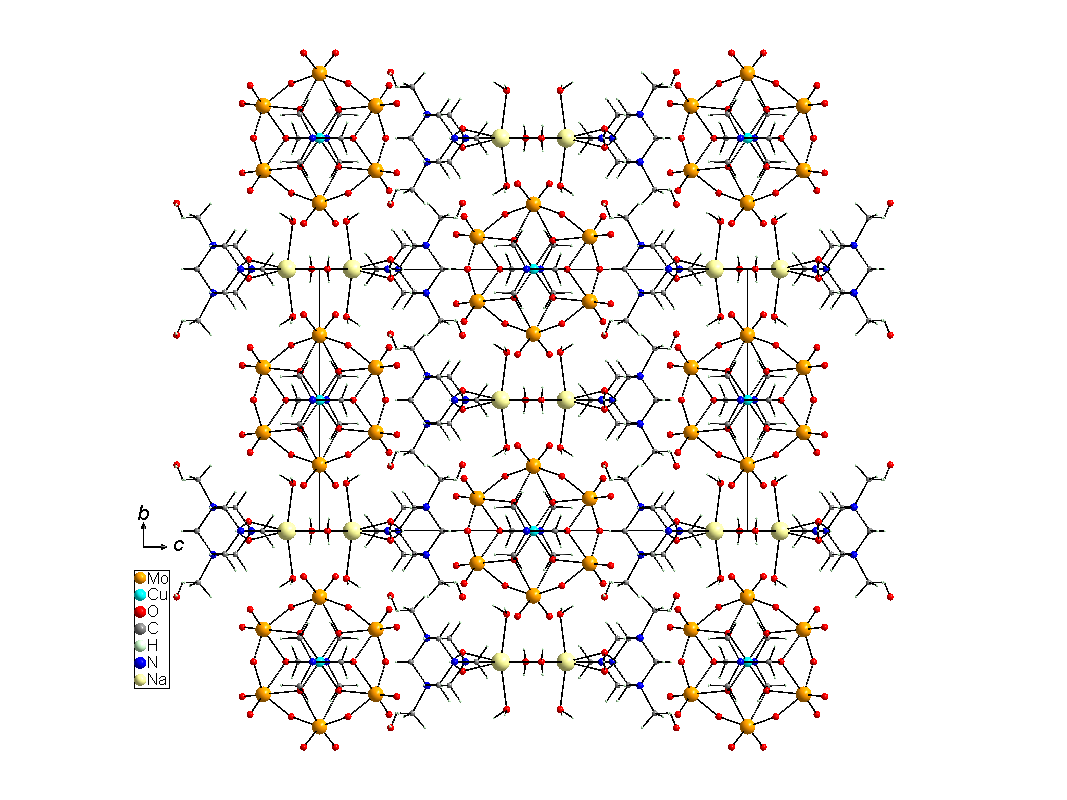


Figure S1. A view along the a-axes of the crystal packing of Cu^II^-POM.

Figure S2. The EDX spectrum of the compound Cu^II^-POM.


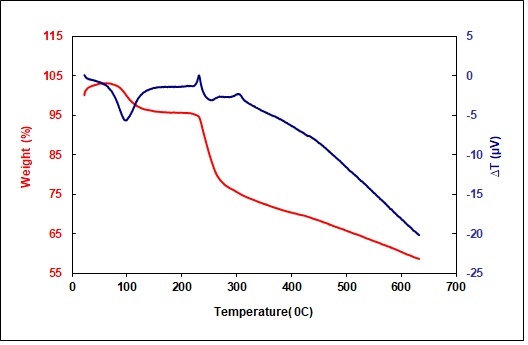


Figure S3. Thermogravimetric analysis (TGA) curve of the Cu^II^-POM.

Figure S4. FT-IR spectra of the fresh and used Cu^II^-POM photocatalyst.


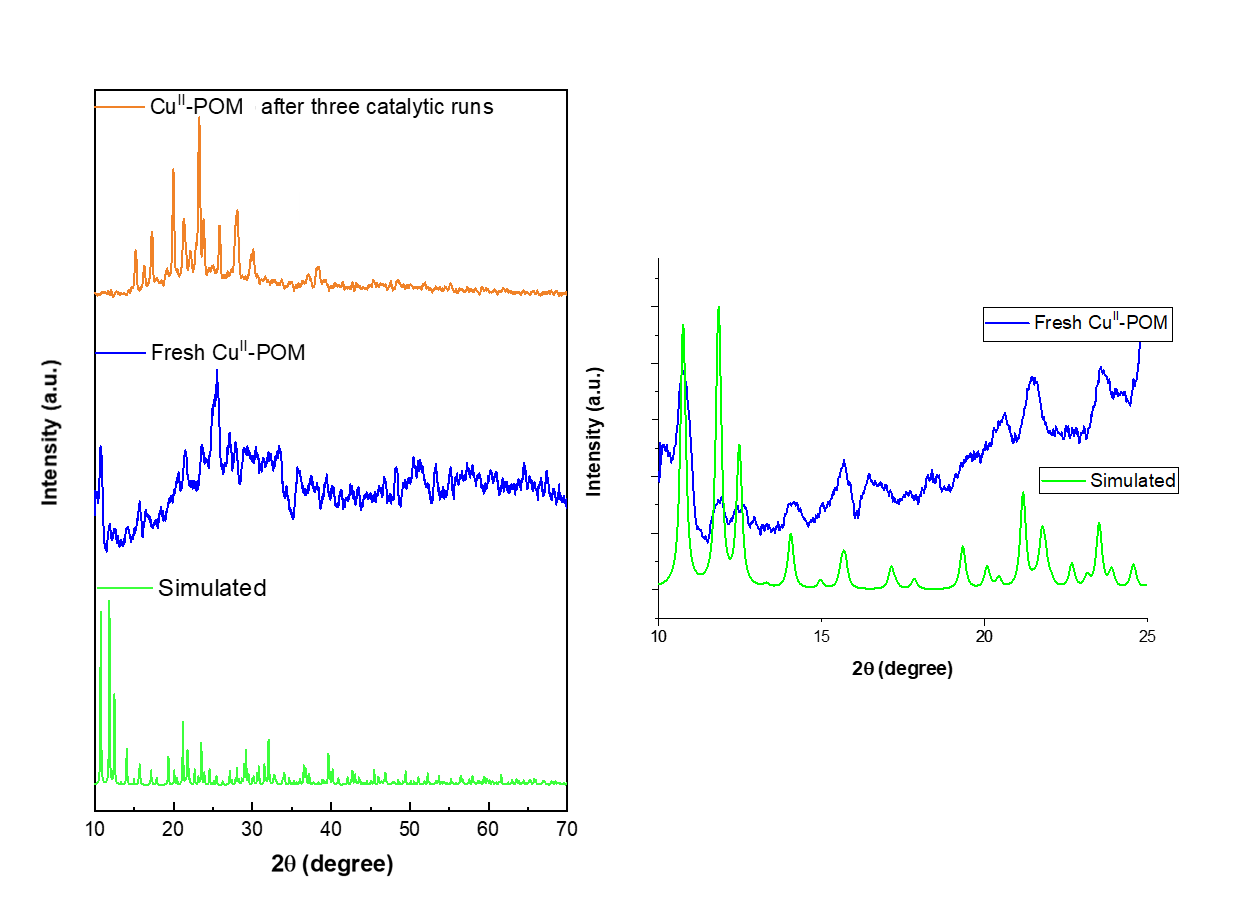


Figure S5. (left) PXRD patterns of the simulated, fresh and used Cu^II^-POM photocatalyst; (right) partially enlarged PXRD patterns of the simulated and fresh Cu^II^-POM between 10° and 25°.

Table S3. Comparison of the catalytic activity of Cu^II^-POM with various catalysts reported in the literature for the Cu-AAC reaction.

| Entry | Catalyst (amount) | Reaction conditions: substrates/solvent/temp./time | Yield (%) | Ref. |
| --- | --- | --- | --- | --- |
| 1 | [CuI_4_(SiW_12_O_40_)(L)]·6H_2_O·2DMF (0.92 mmol) | benzyl azide (1 mmol)/phenylacetylene (2 mmol)/EtOH/80 °C/12 h | 99 | [5] |
| 2 | Cu Wire (50 mmol) | Coumarin azide (0.05 mmol)/mPEG-alkyne (0.05 mmol)/PMDTA (0.05 mmol)/under inert atmosphere/toluene/80 °C/48 h | 92 | [6] |
| 3 | Cu(I)(CH_3_CN)_4_PF_6_ (1 mmol) | Azide-TBA_6_[P_2_W_15_V_3_](2 mmol)/phenylacetylene (0.5 mmol)/DIPEA/70 °C/4 h | 94 | [7] |
| 4 | saCu-2@mpgC_3_N_4_ (1 mmol) | benzyl bromide (1 mmol)/NaN_3_ (1 mmol)/phenylacetylene (1 mmol) /DMF/100-140 °C/30 min | 45 | [8] |
| 5 | Cu-Fe_3_O_4_-PAC (50 mg) | phenylacetylene (1.1 mmol)/NaN_3_ (1.2 mmol)/benzyl bromide (1 mmol)/ r.t./H_2_O/22 h | 99 | [9] |
| 6 | (C_7_H_15_N_4_)_2_ [Co(H_2_O)_6_] [C_6_H_12_N_2_CoMo_6_O_24_]**·**4H_2_O (10 mg) | benzyl chloride (0.55 mmol)/NaN_3_ (0.55 mmol)/phenylacetylene (0.5 mml)/H_2_O/80 °C/2 h | 99 | [10] |
| 7 | Cu^II^-POM (0.00239 mmol) | benzyl chloride (0.55 mmol)/NaN_3_ (0.55 mmol)/phenylacetylene (0.5 mml)/H_2_O/80 °C/8 h/UV | 97 | Present work |

DIPEA= N, N-diisopropylethylamine, PMDTA= N, N, N′, N′, N′-Pentamethyl diethylenetriamine, resorcin[4]arene-based ligand (L).

# References

1. CrysAlis CCD and CrysAlis Red 1.171.38.43, Rigaku Oxford Diffraction, Yarnton, UK. 2015.

2. G.M. Sheldrick, SHELXT - Integrated space-group and crystal-structure determination. Acta Crystallogr. Sect. A: Found. Adv. 71 (2015) 3- 8.

3. G.M. Sheldrick, Crystal structure refinement with SHELXL. Acta Crystallogr. Sect. C: Cryst. Struct. Commun. 71 (2015) 3-8.

4. K. Brandenburg, H. Putz, DIAMOND Version 3.0, Crystal Impact GbR, Bonn, Germany, 2006.

5. Lu, B. B., Yang, J., Che, G. B., Pei, W. Y., & Ma, J. F. Highly Stable Copper(I)-Based Metal-Organic Framework Assembled with Resorcin [4] arene and Polyoxometalate for Efficient Heterogeneous Catalysis of Azide-Alkyne “Click” Reaction. ACS Appl. Mater. Interfaces. **41**, 2628−2636 (2017).

6. López, S., García-Vargas, J. M., García, M. T., Rodríguez, J. F., Gracia, I., & Ramos, M. J. Copper-Containing Catalysts for Azide– Alkyne Cycloaddition in Supercritical CO_2_. *Catalysts* **12**, 194 (2022).

7. Vanhaecht, S., Quanten, T., & Parac-Vogt, T. N. A mild post-functionalization method for the vanadium substituted P_2_W_15_V_3_ Wells– Dawson polyoxometalate based on a copper catalyzed azide–alkyne cycloaddition. *Dalton Trans.* **46**, 10215−10219 (2017).

8. Vilé, G. *et al*. Azide-Alkyne Click Chemistry over a Heterogeneous Copper-Based Single-Atom Catalyst. *ACS Catal.* **12**, 2947−2958 (2022).

9. Aflak, N. *et al*. Copper- on- Magnetically Activated Carbon-Catalyzed Azide-Alkyne Click Cycloaddition in Water. *Catalysts* **12**, 1244−1260 (2022).

10. Yousofvand, A., Amini, M., Hosseinifard, M., & Janczak, J. Synthesis, structure, and catalytic activity of polyoxometalate (C_7_H_15_N_4_)_2_[Co(H_2_O)_6_][C_6_H_12_N_2_CoMo_6_O_24_]·4H_2_O in the azide–alkyne cycloaddition reaction. *Polyhedron*. **236**, 116367 (2023).
